# Substance use risk screening and associated factors among Costa Rican secondary students: a nationally representative analysis

**DOI:** 10.3389/fpubh.2025.1652601

**Published:** 2025-09-22

**Authors:** Andrea Lopez-Soto, Esmeralda Ramirez, Jeancarlo Cordoba, Pablo Montero-Zamora

**Affiliations:** ^1^Department of Kinesiology and Health Education, University of Texas at Austin, Austin, TX, United States; ^2^School of Public Health, University of Costa Rica, San Jose, Costa Rica

**Keywords:** adolescent substance use, CRAFFT screener, Costa Rica, screening tool, violence, national survey

## Abstract

**Background:**

Adolescent substance use (SU) is a significant public health concern in Latin America, however, representative data to guide prevention efforts remain limited. This study examined SU risk and potential associated factors among Costa Rican secondary students using the CRAFFT 2.1 screening tool.

**Methods:**

Data were drawn from the 2021 VI National Survey on Psychoactive Substance Use in the Secondary School Population, a nationally representative sample of 3,524 students (weighted *N* ≈ 354,330). Based on CRAFFT scores, students were classified into low-, medium-, and high-risk SU groups. Multinomial logistic regression analyses assessed associations between SU risk levels and sociodemographic characteristics, SU within the family, sexual activity under SU, and exposure to violence.

**Results:**

Overall, 64.7% of students were classified as low-risk, 26.6% medium-risk, and 8.7% high-risk. Older age, substance use by family members (i.e., smoking and illicit drug use), sexual activity under SU, and exposure to psychological and sexual violence were all associated with increased odds of classification into the high-risk group. Sexual activity under SU was strongly associated with membership in the high-risk group compared to both low- and medium-risk groups.

**Conclusion:**

This study is the first to apply the CRAFFT tool to a nationally representative sample of Costa Rican adolescents, providing critical insights for prevention initiatives in Latin America. Findings emphasize the need for universal multi-level prevention interventions to delay SU initiation and promote prosocial environments that support healthy youth development, ultimately reducing the burden of SU among Costa Rican adolescents.

## 1 Introduction

Adolescence is a critical developmental period characterized by profound biological, social, emotional, and cognitive transformations (1). While this period offers numerous opportunities for learning and growth, it also presents heightened vulnerability to adverse experiences, including exposure to violence, abusive relationships, and substance use (SU) (2). Among these adverse experiences, adolescent SU has emerged as a prevalent and concerning maladaptive coping mechanism often observed in response to challenges associated with this developmental stage (3, 4). As noted by Connolly (5), adolescents often engage in SU to achieve a sense of calm or relaxation, to escape distressing thoughts or memories, to cope with symptoms of depression, or to seek pleasurable or novel experiences.

Adolescent SU is associated with a range of adverse outcomes that might extend into adulthood. These include health-related, psychological, legal, and criminal justice consequences (6). For instance, in the United States (U.S.), a significant proportion of adults (~90%) with substance use disorders (SUDs) report initiating SU during adolescence, underscoring the enduring impact of early substance involvement (7, 8). Given these long-term implications, adolescent SU continues to be recognized as a pressing global public health concern. According to an analysis of the 2021 Global Burden of Disease Study (9), approximately 5 million new cases and 10 million existing cases of SUDs were reported among adolescents worldwide. The findings revealed (a) a higher prevalence of SUDs among males compared to females; (b) a significant upward trend in the burden of adolescent SUDs in high-income North American and tropical Latin American countries; and (c) a marked increase in both prevalence and incidence in the Caribbean and Central America—indicating a rapidly escalating public health concern in these regions.

In Central America, countries such as Costa Rica exemplify the urgent need to address adolescent SU. National data from 2018 on individuals aged 15 to 24 indicate that alcohol was the most commonly used substance across the lifespan (59.6%), followed by cigarettes (24.6%), marijuana (18.9%), and other illicit drugs (3.5%) (10). Similarly, findings from the Fifth National Survey on Psychoactive Substance Use in the Secondary School Population, also conducted in 2018, reported comparable patterns among Costa Rican high school students. Alcohol remained the most frequently consumed psychoactive substance (69.9%), followed by cigarettes (13.1%), marijuana (9.4%), and other illicit drugs (~3%) (11). These data reflect a consistent pattern of early substance initiation, highlighting the critical need for targeted, context-specific prevention and intervention strategies within the country.

Implementing validated screening tools is crucial for the early detection and intervention of adolescent SU. These tools—also referred to as instruments—are valuable for identifying the presence, severity, and specific nature of substance-related problems, as well as for determining which adolescents may require more comprehensive clinical assessment (12). Current SU literature includes several screening instruments specifically designed for adolescents. Notably, the *Car*, *Relax Alone*, *Forget Friends*, and *Trouble* (CRAFFT) screening tool (13) is widely used and well-validated for adolescent populations (14). Additionally, the National Institute on Drug Abuse (NIDA) recommends two brief online tools for assessing SUDs risk among adolescents aged 12 to 17: (a) the Brief Screener for Tobacco, Alcohol, and Other Drugs (BSTAD) (15); and (b) the Screening to Brief Intervention tool (S2BI) (16). Other commonly used instruments include the Personal Experience Screening Questionnaire (PESQ) (17), and the Substance Abuse Subtle Screening Inventory—Adolescent, Second Edition (SASSI-A2) (18).

The CRAFFT is a screening tool specifically designed to assess the risk of adolescent SU and has also been used to identify potential alcohol- and drug-related problems (19). Developed by Knight et al. (13), the CRAFFT comprises nine items, incorporating questions derived from previously validated instruments such as the Problem-Oriented Screening Instrument for Teenagers (POSIT) (20), Relax, Alone, Friends, Family, Trouble (RAFFT) (21), and Drug and Alcohol Problems (DAP) (22).

The CRAFFT is named after a mnemonic that reflects six core questions assessing behaviors related to driving under the influence (i.e., Car), using substances to relax (i.e., Relax), using alone (i.e., Alone), memory loss (i.e., Forget), concern from others for your SU (i.e., Friends), and getting into problems due to use (i.e., Trouble). These are typically preceded by three initial questions about the past 12 months of substance use (23). The CRAFFT has consistently demonstrated strong psychometric properties, with sensitivity for detecting SU, misuse, or disorders ranging from 0.61 to 0.98, and specificity between 0.73 and 0.97 across multiple validation studies (24–26). Importantly, comparable results have been reported in Spanish-speaking populations, including studies conducted in Argentina (27, 28), Colombia (29), Mexico (30), and Spain (31). However, no validation or exploratory studies using the CRAFFT have been conducted in Central American countries, such as Costa Rica.

The CRAFFT scoring system provides valuable insight into the severity of SU behaviors and associated clinical outcomes. A score of 2 or higher is widely recognized as the optimal cutoff for identifying SUDs among adolescents aged 14 to 18. It has been shown to predict DSM-5 SUD diagnoses across all levels of severity (25–27, 32, 33). Notably, higher CRAFFT scores are strongly associated with a greater likelihood of severe SU problems. For example, scores of 4, 5, and 6 correspond to progressively greater probabilities—54, 70, and 100%, respectively—of an adolescent meeting the criterion for moderate to severe SUDs (26).

To help guide clinical decision-making, the CRAFFT developers introduced a risk-based classification framework aligned with score thresholds and reported behaviors (23). Adolescents with no SU or related risk indicators in the past 12 months (e.g., a negative response to the “Car” item) fall into the “low-risk” category. The “medium-risk” category includes individuals with either substance-related safety concerns despite no recent use or those reporting recent use without the presence of any risk indicator (e.g., using to relax or when alone). The “high-risk” category comprises adolescents who report past-year use along with two or more risk indicators, underscoring the need for comprehensive clinical evaluation and targeted intervention (23).

Most research on the CRAFFT screening tool has focused on its diagnostic accuracy for identifying SUDs and its psychometric properties (34–39), with evaluations most commonly conducted in primary care settings (40–42). However, an emerging body of literature has examined its application in schools to categorize adolescent SU risk and support targeted prevention efforts. For example, Alayan and Shell implemented CRAFFT screening in school health services led by nurse practitioners, using the developers’ three-level risk framework (i.e., low, moderate, and high risk) to guide brief interventions and referrals. Their findings emphasize the feasibility of integrating CRAFFT into routine school-based healthcare and the value of tailoring interventions according to risk severity (43). Similarly, Falck et al. surveyed nearly 4,000 high school students across 16 districts and found that one-third screened positive for problematic use (CRAFFT ≥2), with 14% showing signs of dependence. Higher CRAFFT scores were associated with use of a greater number of substances. Substance use risk was more prevalent among 12th graders compared to 11th graders, and among older adolescents, boys were more likely than girls to present signs of dependence—highlighting the substantial burden of SU in school populations (44).

Expanding on this work, Agley et al. (45) analyzed CRAFFT data from over 25,000 Indiana students to assess the spectrum of SU severity and examine predictors of risk levels among substance-using adolescents. They identified three severity categories: non-problematic (scores 0–1), problematic (2–3), and dependent (4–6), with nearly 20% of students falling into the dependent category. Risk factors such as academic failure, low school commitment, and association with antisocial peers were associated to greater SU severity, whereas interaction with prosocial peers emerged as a protective factor. Notably, these associations varied across risk levels. Poly-substance use emerged as a consistent predictor of both problematic and dependent use (45). Together, these studies exhibit the consistent application of CRAFFT-defined risk thresholds in school settings and underscore the tool’s utility in identifying varying levels of SU severity among adolescents. However, there is still limited understanding of which etiologic factors most strongly predict adolescents’ classification into the tool’s distinct SU risk categories.

Regarding specific etiologic factors (i.e., risk and protective factors) associated with adolescent SU and misuse, the literature has documented positively correlated individual- and family-level risk factors. For instance, at the *individual level*, research has identified risk factors such as child maltreatment (e.g., exposure to sexual, physical, or psychological violence) (46) and engaging in sexual activity while under the influence of substances (47–49). Further, from a *family-level* perspective, parental SU has also been shown to significantly increase the likelihood of SU and misuse among their children (50, 51). While these etiological factors are well-established predictors of SU initiation and general problematic use, their specific relationship with CRAFFT-defined risk levels remains unclear. In particular, little is known about how these individual- and family-level etiological factors influence the likelihood of being classified as low- vs. medium-risk or medium- vs. high-risk for adolescent SU and misuse. Understanding these associations is crucial for developing multi-level prevention initiatives tailored to specific contexts and populations.

Despite the widespread use of the CRAFFT screening tool across various adolescent populations globally, a significant gap remains in its application within Central American countries, such as Costa Rica. Furthermore, as mentioned earlier, while the CRAFFT tool provides a robust framework for categorizing substance use risk levels (low, medium, and high), limited research has explored the specific etiologic factors associated with these risk levels. Investigating potential predictors or etiologic factors—such as sociodemographic characteristics, family history of substance use, engagement in risky behaviors like sexual activity under the influence, and exposure to violence—could offer critical insights into the drivers that escalate SU to misuse. Addressing this gap would not only provide a more nuanced understanding of the vulnerabilities contributing to SU and its severity but also help tailor interventions more effectively to the specific needs of countries like Costa Rica.

To our knowledge, no prior studies in Costa Rica-or Central America-have utilized the CRAFFT screening tool with high school-attending students, nor investigated the potential predictors associated with the risk levels identified by this tool. Therefore, this study aims to (a) examine responses to the CRAFFT screening tool among Costa Rican secondary school students, (b) classify substance use risk into low, medium, and high categories based on internationally recommended CRAFFT thresholds and describe their prevalence, and (c) analyze the associations between CRAFFT risk levels and potential predictors, including sociodemographic factors, family history of SU, sexual activity under the influence of substances, and exposure to various types of violence (i.e., physical, psychological, and sexual). To achieve our study aims, we used data from a nationally representative survey conducted by a Costa Rican governmental institution, the VI National Survey on Psychoactive Substance Use in the Secondary School Population (VI-NSPSSU) (52). Further details about the VI-NSPSSU are provided in the Methods section.

## 2 Methods

### 2.1 Study design and participants

The present study used data from the VI National Survey on Psychoactive Substance Use in the Secondary School Population (VI-NSPSSU) (52), conducted in 2021 by the Costa Rican Institute on Alcoholism and Drug Addiction (IAFA). IAFA is a governmental institution responsible for the study, prevention, treatment, and rehabilitation of addiction related to alcohol, tobacco, and other licit or illicit substances (53). The VI-NSPSSU was a nationally representative, cross-sectional survey of secondary students in Costa Rica. It collected data on the use of psychoactive substances and associated risk factors (e.g., demographics, CRAFFT scores, violence exposure, family SU). Notably, the VI-NSPSSU was the first version of this national survey to include the CRAFFT screening tool (52).

According to IAFA, a multi-stage (i.e., two stages) stratified area probability sample (54) of 3,524 individuals (i.e., elements) selected across 60 schools (i.e., clusters or secondary sampling units) within 10 geostatistical areas (i.e., primary sampling units) was randomly selected from a preestablished school system sample frame. In the first stage, schools were classified into 10 strata based on IAFA’s administrative regions (e.g., Central East, Brunca). Within each region, schools were further stratified based on whether they offered education from 7th to 11th grade or from 7th to 12th grade, resulting in 20 strata in total. Schools were then selected within each stratum using probability proportional to size without replacement. In the second stage, one classroom section per grade level was randomly selected within each chosen school, and all students present in the selected sections were invited to participate in the study (52).

Sample weights—statistical adjustments developed by the IAFA statistical team and applied by our team for this study—were used to ensure the sample accurately reflects the national population by correcting for unequal selection probabilities and non-response. After applying these weights, the final study sample consisted of 3,524 individuals, representing approximately 354,330 Costa Rican adolescents enrolled in the national education system. Of these, 185,912 participants (54.4%) were identified as female. Participants ranged in age from 11 to 20 years (M = 15.22, SD = 1.66). Nearly 239,500 students (67.6%) attended high schools in urban districts. Additionally, 38,424 students (11.3%) reported engaging in some form of paid work while attending school, such as hourly or occasional jobs.

### 2.2 Procedures

Data collection was conducted using an online survey (~ 55 min). The survey was accessible via computer, tablet, or smartphone through a system enabled by the school. Participants who provided informed consent proceeded to complete the survey. To conduct the present study, the first author submitted a formal request to IAFA for access to the de-identified dataset from the VI-NSPSSU. This request was approved. Additionally, all study procedures were reviewed and approved by the Institutional Review Board at the corresponding author’s affiliated institution prior to the commencement of the research.

### 2.3 Measures

#### 2.3.1 Substance use risk

Participants’ risk level for SU was assessed using the CRAFFT 2.1 version screening tool (Car, Relax, Alone, Forget, Friends, Trouble) (23). The CRAFFT 2.1 consists of two parts (a) Part A assesses SU frequency by asking participants to report the number of days in the past 12 months on which they consumed more than “a few sips” of alcohol, used cannabis or synthetic cannabinoids, or used another substance to get high; and (b) Part B includes six dichotomous items (*yes* = 1, *no* = 0) assessing substance-related experiences (e.g., riding in a car driven by someone, including oneself, under the influence of substances; using substances to relax or feel better; see Table 1 for item details). Responses to Part B were summed to produce a total CRAFFT score ranging from 0 to 6. In the present sample, Cronbach’s alpha for Part B was *α* = 0.75.

**Table 1:** CRAFFT screening tool frequencies of item responses and scores (*N* = 354,330).

Item	Population
	% Yes
*Part A*	
1. Drink more than a few sips of beer, wine or any drink containing alcohol in the last year?^a^	26.2
2. Use any marijuana (weed, oil, or hash by smoking, vaping, or in food) or “synthetic marijuana” (like “K2,” “Spice”) in the last year?^a^	4.1
3. Use of anything else to get high (like other illegal drugs, prescription or over-the-counter medications, and things that you sniff, huff or vape) in the last year?^a^	7.4
*Part B*	
C. Have you ever ridden in a CAR driven by someone (including yourself) who was “high” or had been using alcohol or drugs?	16.1
R. Do you ever use alcohol or drugs to RELAX, feel better about yourself, or fit in?	4.8
A. Do you ever use alcohol or drugs to while you are by yourself, or ALONE?	6.3
F. Do you ever FORGET things you did while using alcohol or drugs?	4.8
F. Do your FAMILY or FRIENDS ever tell you that you should cut down on your drinking or drug use?	3.6
T. Have you ever gotten into TROUBLE while you were using alcohol or drugs?	2.0
*CRAFFT scores*	% Frequency
0	79.0
1	12.3
2	3.7
3	2.5
4	1.8
5	0.5
6	0.3

^a^% Yes represents consumption of 1 or more days.

**Figure 1:**
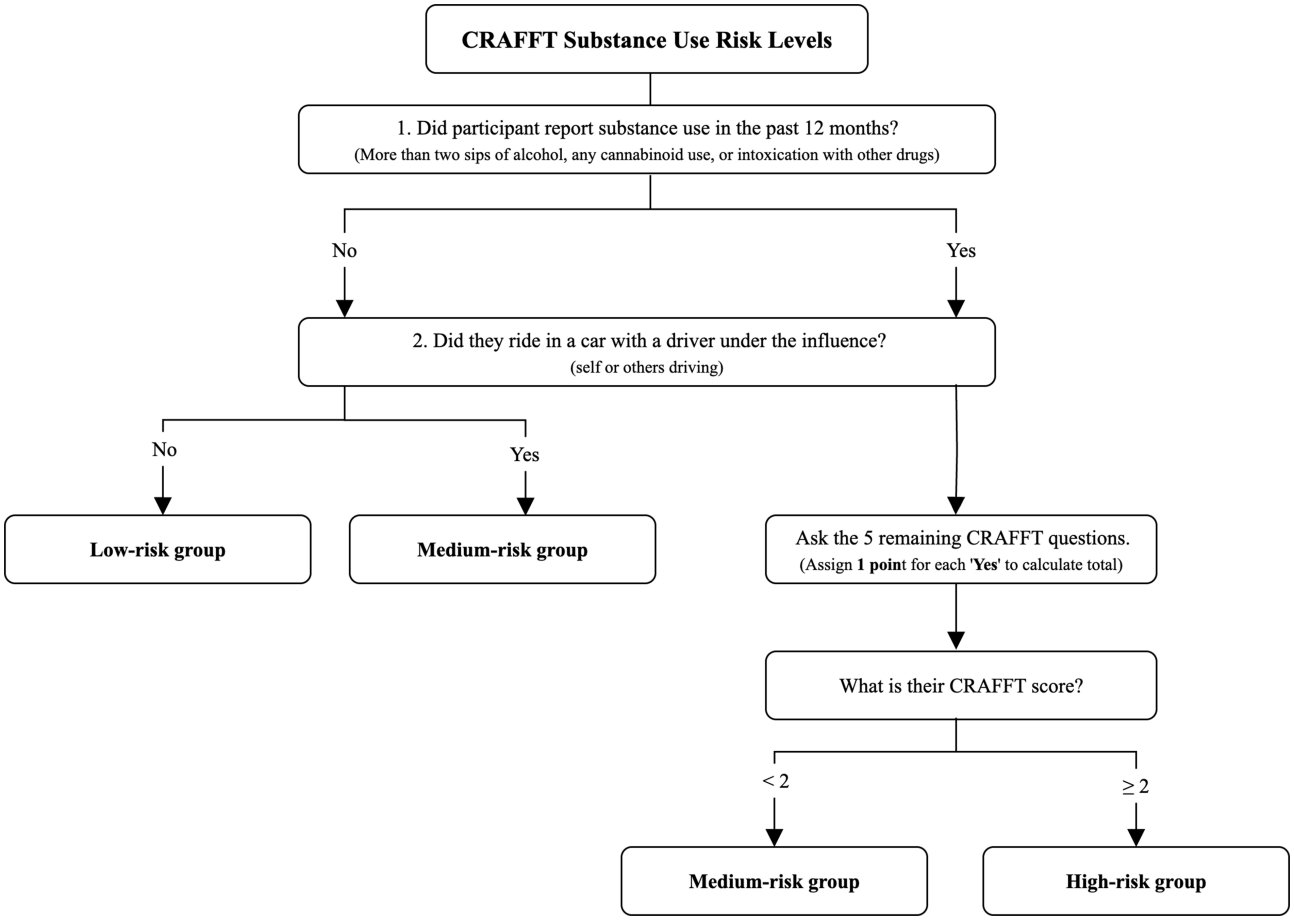
Decision tree for classifying adolescent substance use risk using the CRAFFT 2.1.

Risk categories were defined using the CRAFFT 2.1 screening tool and operationalized based on both SU behaviors reported in the past 12 months and CRAFFT scores, following the developers’ cut-off criteria. As shown in Figure 1, participants were classified as low-risk if they reported (1) no SU in the past year and (2) never driving under the influence of alcohol or drugs, nor riding in a vehicle with an impaired driver. The medium-risk group included participants who met one of two conditions (1) first, those who did not report SU but had ridden in a vehicle with a driver (either themselves or another person) under the influence and (2) second, those who had engaged in SU in the past 12 months but scored less than two on the CRAFFT, indicating fewer risk-related behaviors. Finally, participants were classified as high-risk if they both reported SU within the past year and had a CRAFFT score of 2 or higher, reflecting higher levels of substance-related problems or risks. These categories integrate behavioral indicators and validated screening scores to provide a nuanced classification of SU risk.

#### 2.3.2 Sociodemographic

Participants reported their sex (*male* = 0, *female* = 1), age group (*11*–*14 years* = 0, *15*–*17 years* = 1, *18*–*20 years* = 2), and whether they engaged in paid work while attending school, such as hourly or occasional employment (*yes* = 1, *no* = 0). To determine the degree of urbanization of each participant’s high school location (*rural* = 1*, urban* = 0), we identify the districts where the schools are situated and utilize the district’s urbanization degree rating from the Costa Rican National Institute of Statistics and Census (55).

#### 2.3.3 Substance use within the family

Exposure to substance use within the family was assessed using three items (1) binge drinking in the household (e.g., ‘*Does anyone in your household drink excessive alcohol or get drunk frequently?’*); (2) smoking in the household (e.g., *‘Does anyone in your household smoke tobacco or use nicotine?’*); and (3) illegal drug use in the household (e.g., *‘Does anyone in your household use any of the following substances: marijuana, cocaine, crack, hallucinogens, or ecstasy?’*). Responses to each item were coded dichotomously (*yes* = 1, *no* = 0).

#### 2.3.4 Sexual activity under substance use

Sexual activity under the influence of substance use was measured with a single item: ‘*Have you had sex while under the influence of alcohol or other drugs*?’ Responses were coded dichotomously (*yes* = 1, *no* = 0).

#### 2.3.5 Violence exposure

Violence exposure was measured using three items related with (1) physical violence (e.g., *‘Have you ever experienced physical aggression that you have not been able to overcome?’*), (2) psychological violence (e.g., *‘Have you ever experienced psychological aggression that you have not been able to overcome?’*), and (3) sexual violence (e.g., *‘Have you ever experienced sexual aggression that you have not been able to overcome?’*). Responses to each item were coded dichotomously (*yes* = 1, *no* = 0).

### 2.4 Data analysis

All data analyses were conducted using Stata 17’s SVY command (which accounts for the complex survey design, including clustering, stratification, and sampling weights, to produce accurate standard errors and population estimates) (56). First, following the CRAFFT 2.1 developers’ guidelines, participants were categorized into three risk groups—low, medium, and high—based on established cut-off scores. Specifically, those with a CRAFFT score of 0 and no SU in the past 12 months were classified as low-risk. Participants with a score of 1 who either reported no SU but had been a passenger in a vehicle with an impaired driver, or who reported SU but had a CRAFFT score below 2, were classified as medium-risk. Those with a CRAFFT score of 2 or greater were classified as high-risk (see Figure 1). This operationalization allowed for the examination of risk groups as categorical variables in subsequent analyses.

Second, we conducted descriptive analyses of each group of participants based on their SU risk levels, in terms of their sociodemographic characteristics and potential predictors (i.e., exposure to substance use within the family, risky sexual activity, and exposure to violence). Third, we used the Rao-Scott adjusted chi-squared test to assess differences across SU risk groups based on sociodemographic characteristics and potential predictors, accounting for the complex survey design, including stratification, clustering (PSUs), and weighting. Finally, we conducted a series of multinomial logistic regressions (57, 58) to examine associations between substance use risk categories (i.e., dependent variable) and potential predictors (i.e., independent variables). For each multinomial regression model, relative risk ratios (RRRs) and confidence intervals (CIs) were calculated and interpreted as the likelihood of belonging to a specific risk category compared with a reference category (e.g., High vs. Low) (59). Multicollinearity within the models was assessed by calculating variance inflation factors (VIFs) from a linear regression model in which CRAFFT SU risk categories were treated as a continuous outcome and including the same independent variables (60). We examined the VIF values to confirm that they fell below the commonly accepted threshold of 5 (61).

## 3 Results

### 3.1 CRAFFT item frequencies and scores

Table 1 presents participants’ responses to each of the nine CRAFFT items, along with the percentage of students who responded affirmatively to each. Overall, 26.2% of the sample reported alcohol use in the past year, 4.1% reported using marijuana or hashish, and 7.4% reported using another type of substance. Among the six CRAFFT risk items (Items 4–9), the highest rate of endorsement was for Item 4 (i.e., *‘Have you ever ridden in a CAR driven by someone [including yourself] who was “high” or had been using alcohol or drugs?’*), with 16.1% responding “*yes*.” The lowest was for Item 9 (*‘Have you ever gotten into trouble while drinking alcohol or taking any kind of drug?’*), endorsed by 2.7% of participants. The mean CRAFFT score was 0.38 (*SD* = 0.92; range = 0–6). The most frequently observed scores were 0 (79.0%) and 1 (12.3%).

### 3.2 Substance use risk levels and characterization

Table 2 provides the distribution of sociodemographic characteristics across the three levels of SU risk, as defined by the cut-off scores established by the CRAFFT developers (i.e., Low = 0, Medium = 1, and High ≥2). Most students (64.7%) were classified as low-risk, followed by 26.6% at medium-risk, and 8.7% at high-risk. Statistically significant differences were observed across risk levels for age, substance use within the family (i.e., binge drinking, smoking, and illegal drug use), sexual activity under substance use, and prior exposures to physical, psychological, and sexual violence (all *p* values < 0.05). In contrast, no significant differences were identified based on sex, the degree of urbanization of the high school, or engagement in paid employment while attending school. Regarding age, participants aged 11 to 14 were predominantly classified as low-risk, with a notably lower representation in the high-risk group. In contrast, students aged 15 to 20 were predominantly classified in the high-risk group.

**Table 2:** CRAFFT substance use risk levels by sociodemographic characteristics, family substance use, sexual behavior, and exposure to violence among Costa Rican adolescents, 2021.

Variables		Levels of risk			
	Total sample	Low	Medium	High	*p*-value^*^
Total	100.0%	64.7%	26.6%	8.7%	
Sex					
Men	45.6%	45.5%	46.7%	42.4%	0.598
Women	54.4%	54.5%	53.3%	57.6%	
Age					
11–14	38.4%	46.1%	27.9%	13.7%	<0.001
15–17	52.4%	47.8%	58.3%	68.6%	
18–20	9.2%	6.1%	13.9%	17.7%	
Rural high school					
No (Urban)	68.8%	67.7%	70.1%	72.7%	0.463
Yes	31.2%	32.3%	29.9%	27.3%	
Work while studying					
No	88.9%	90.0%	87.4%	84.7%	0.176
Yes	11.1%	10.0%	12.6%	15.3%	
Substance use within the family					
Binge drinking in the family					
No	89.1%	93.1%	81.8%	81.9%	<0.001
Yes	10.9%	6.9%	18.2%	18.1%	
Smoking in the family					
No (Ref)	83.5%	87.9%	76.0%	73.2%	<0.001
Yes	16.6%	12.1%	24.0%	26.8%	
Illegal drug use in the family					
No (Ref)	92.5%	95.6%	88.0%	80.5%	<0.001
Yes	7.5%	4.4%	12.0%	19.5%	
Sexual activity under substance use					
No	95.5%	99.0%	97.1%	65.1%	<0.001
Yes	4.5%	1.0%	2.9%	34.9%	
Violence					
Physical					
No	96.8%	96.8%	94.0%	89.2%	0.007
Yes	3.2%	3.2%	6.0%	10.8%	
Psychological					
No	81.7%	86.9%	77.9%	54.9%	<0.001
Yes	18.3%	13.1%	22.1%	45.1%	
Sexual					
No	94.6%	97.2%	93.6%	77.4%	<0.001
Yes	5.4%	2.8%	6.4%	22.6%	

Low-Risk, no use in the past 12 months and answers “No” to the CAR (item 4) question. Medium-Risk, no use in the past 12 months and answers “No” to the CAR question OR Any use in the past 12 months and CRAFFT score of 0 or 1. High Risk, any use in the past 12 months and have a CRAFFT total score of 2 or more. ^*^Rao-Scott adjusted chi-squared test, *p* < 0.05.

In terms of substance use within the family, binge drinking was reported by 18.2% of adolescents in the medium-risk group and 18.1% in the high-risk group, compared to just 6.9% in the low-risk group. A similar pattern emerged for family smoking, with prevalence rates of 24.0 and 26.8% in the low-risk and high-risk groups, respectively, versus 12.1% in the low-risk group. Reports of illegal drug use within the family followed this trend as well, with 12.0% of adolescents in the medium-risk group and 19.5% in the high-risk group endorsing this experience, compared to only 4.4% in the low-risk group.

Finally, sexual activity under SU was more frequently reported among adolescents in the high-risk group (34.9%) compared to only 1.0% in the low-risk group. Similarly, significant differences were observed across risk levels of violence exposures, including physical, psychological, and sexual forms. Adolescents classified in the high-risk group reported the highest prevalence of all three types of violence exposure, (a) physical (10.8%), (b) psychological (45.1%), and (c) sexual (22.6%).

### 3.3 Multinomial logistic regression findings

#### 3.3.1 Medium-risk vs. low-risk

Adolescents aged between 15 and 17 years had significantly greater odds of being classified in the medium-risk group compared to the low-risk group [RRR = 1.87, 95% CI (1.33, 2.64)], as did those aged 18–20 years [RRR = 3.13, 95% CI (1.43, 6.83)]. Binge drinking [RRR = 1.99, 95% CI (1.14, 3.50)], smoking [RRR = 1.74, 95% CI (1.25, 2.42)], and illegal drug use [RRR = 2.04, 95% CI (1.07, 2.64)] significantly predicted medium-risk group membership compared to low-risk. In addition, exposure to sexual violence was associated with increased likelihood of belonging to the medium-risk group [RRR = 1.85, 95% CI (1.11, 3.09)] compared to the low-risk group (see Table 3).

**Table 3:** Multinomial logistic regression model results.

	Risk categories					
	Medium vs. Low		High vs. Low		High vs. Medium	
Variables	RRR	95% CI	RRR	95% CI	RRR	95% CI
Sex						
Men (Ref)	-	-	-	-	-	-
Women	0.87	0.69–1.08	0.76	0.48–1.20	0.88	0.58–1.32
Age						
11–14 (Ref)	-	-	-	-	-	-
15–17	1.87^***^	1.33–2.64	3.49^***^	2.04–6.00	1.87^*^	1.00–3.49
18–20	3.13^**^	1.43–6.83	4.45^**^	1.73–11.43	1.42	0.60–3.34
Rural high school						
No (Ref)	-	-	-	-	-	-
Yes	1.05	0.76–1.44	1.19	0.71–2.00	1.14	0.73–1.76
Work while studying						
No (Ref)	-	-	-	-	-	-
Yes	0.99	0.61–1.61	0.88	0.37–2.07	0.88	0.42–1.84
Substance use within the family						
Binge drinking in the family						
No (Ref)	-	-	-	-	-	-
Yes	1.99^**^	1.13–3.50	1.42	0.70–2.90	0.71	0.38–1.35
Smoking in the family						
No (Ref)	-	-	-	-	-	-
Yes	1.74^**^	1.25–2.42	1.93^**^	1.18–3.16	1.11	0.67–1.85
Illegal drug use in the family						
No (Ref)	-	-	-	-	-	-
Yes	2.04^*^	1.07–4.18	2.64^***^	1.53–4.58	1.25	0.59–2.67
Sexual activity under SU						
No (Ref)	-	-	-	-	-	-
Yes	2.04	0.88–4.72	30.72^***^	12.83–73.55	15.08^***^	7.19–31.67
Violence						
Physical						
No (Ref)	-	-	-	-	-	-
Yes	1.22	0.60–2.49	0.91	0.25–3.32	0.75	0.19–2.86
Psychological						
No (Ref)	-	-	-	-	-	-
Yes	1.46	0.99–2.17	3.96^***^	2.31–6.80	2.71^***^	1.52–4.84
Sexual						
No (Ref)	-	-	-	-	-	-
Yes	1.85^***^	1.11–3.09	4.78^***^	2.89–7.89	2.58^**^	1.27–5.25

RRR, relative risk ratios; 95% CI, 95% confidence interval; Ref, reference; SU, substance use.

*p < 0.05;*p ≤ 0.01;***p ≤ 0.001.

#### 3.3.2 High-risk vs. low-risk

As shown in Table 3, adolescents in the high-risk group exhibited even stronger associations with several potential predictors than those in the low-risk group. For instance, individuals aged 15–17 [RRR = 3.49, 95% CI (2.04–6.00)] and 18–20 [RRR = 4.45, 95% CI (1.73, 11.43)] exhibited significantly greater odds of high-risk classification. Compared to participants in the low-risk group, family smoking [RRR = 1.93, 95% CI (1.18, 3.16)], illegal drug use [RRR = 2.64, 95% CI (1.53, 4.58)], and sexual activity under SU [RRR = 30.72, 95% CI (12.83, 73.55)] were significantly associated with a high-risk SU status. Additionally, exposure to psychological [RRR = 3.96, 95% CI (2.31, 6.80)] and sexual violence [RRR = 4.78, 95% CI (2.89, 7.89)] significantly increased the likelihood of high-risk classification relative to those within the low-risk group.

#### 3.3.3 High-risk vs. medium-risk

When comparing adolescents in the high-risk group to those in the medium-risk group, individuals aged 15–17 demonstrated significantly higher odds of high-risk classification [RRR = 1.87, 95% CI (1.00, 3.49)]. Further, adolescents reporting sex under SU influence evidenced a greater likelihood of being in the high-risk group compared to the medium-risk group [RRR = 15.08, 95% CI (7.19, 31.67)]. Exposure to psychological violence also remained significantly and positively associated with a high-risk group membership [RRR = 2.71, 95% CI (1.52, 4.84)], as did exposure to sexual violence [RRR = 2.58, 95% CI (1.27, 5.25)].

## 4 Discussion

The present study aimed to explore items from the CRAFFT screening tool among Costa Rican secondary school students, categorize SU risk levels, and examine the association between SU risk levels and potential predictors (i.e., etiological factors). Findings suggest that the majority of students (~65%) were classified as having a low risk of SU, based on the CRAFFT screening thresholds. Approximately one-third of participants were identified as having medium or high levels of substance use risk. SU within the family, risky sexual behavior, and exposure to psychological and sexual violence significantly increased the odds of being classified in the medium or high SU risk categories, compared to adolescents who reported no SU in the past year (i.e., low-risk). Notably, engaging in sexual activity under the influence of substances was strongly associated with classification in the high-risk group, even when compared to both the low- and medium-risk groups. These findings underscore the importance of addressing SU and potential etiologic factors using a multilevel approach (e.g., individual and family levels) when designing preventive interventions for SU among Costa Rican adolescents.

Alcohol was the most commonly used substance among Costa Rican adolescents in the past year, consistent with national surveys dating back to 2006 (11). This finding aligns with a systematic review of over 70 international studies identifying alcohol as the most prevalent substance across experimental, single-substance, and polysubstance use (62). Findings also showed that Costa Rican adolescents had lower mean CRAFFT scores (*M* = 0.38) compared to those reported in other studies conducted in the U.S. (*M* = 0.66) (63) and Spain (*M* = 1.60) (31), indicating comparatively lower substance use risk. Additionally, the proportion of Costa Rican adolescents classified in the high-risk group (~9%) was notably lower than the prevalence observed in studies conducted in Argentina (also collected before the COVID-19 pandemic), where the proportion of this high-risk group exceeded 29% (27, 28).

A plausible explanation for these comparatively lower CRAFFT scores and risk classifications lies in the timing of data collection. Specifically, the Costa Rican data were gathered in 2021, amid the COVID-19 pandemic, during which national social restrictions—including school closures and limitations on public gatherings—remained in place until April 2022 (64). By contrast, the comparative studies from the U. S, Spain, and Argentina were conducted prior to the pandemic, under social conditions that may have permitted greater opportunities for adolescent substance use.

Declines in adolescent SU prevalence have been documented internationally during the COVID-19 pandemic period-compared to previous years. For instance, an analysis of the U.S. Youth Risk Behavior Survey data revealed decreases in alcohol use (from 29.2 to 22.7%), marijuana use (from 21.7 to 15.8%), and binge drinking (from 13.7 to 10.5%) between 2019 and 2021. Despite these declines, approximately one-third of U.S. students continued to report past-30-day SU, with polysubstance use remaining prevalent (~35%) (65). These findings suggest that pandemic-related disruptions, such as social distancing and heightened family presence, may have temporarily limited adolescents’ access to substances and opportunities for use. It is important to note, however, that adolescent SU in the U.S. had been on a downward trajectory for over a decade prior to the pandemic (65).

It is also critical to acknowledge that pre-pandemic CRAFFT data for Costa Rica are unavailable, as this study represents the first nationally representative survey employing this screening tool in this context. Although pandemic-related social conditions may partly explain the observed lower risk scores, caution is warranted in their temporal interpretation as we lack other comparative data. Ongoing surveillance of adolescent SU trends under typical social conditions will be essential to determine whether these findings reflect enduring behavioral patterns or transient effects resulting from public health restrictions.

Regarding the association between CRAFFT-defined risk groups and potential predictors, our findings demonstrate that the likelihood of being classified in the medium- or high-risk category significantly increases with age, compared to the low-risk group (i.e., no SU in the past year). This pattern aligns with findings from Montero-Zamora et al. (10), who also identified older age as a significant predictor of SU risk among Costa Rican youth. One possible explanation for this age-related increase in risk is that older adolescents may perceive greater ease of access to psychoactive substances. This perceived ease of access is partly related to the legal drinking age in Costa Rica, which is 18 years; notably, our sample included individuals aged 18 to 20 who have legal access to alcohol despite still being enrolled in school. Therefore, since some classmates are of legal age to purchase alcoholic beverages, they may bring alcohol to social events and share it with their underage friends, thereby potentially facilitating access.

Within the family, SU—including binge drinking, smoking, and illegal drug use—was associated with an increased likelihood of being classified in a higher risk group compared to the low-risk group. This finding is consistent with prior research identifying parental SU as a robust predictor of adolescent SU. For example, a meta-analysis by McGovern et al. (66) found that both maternal and paternal SU significantly increase the likelihood of alcohol-related problems in adolescents. In our study, family binge drinking specifically differentiated individuals in the medium-risk group from those in the low-risk group. In contrast, family smoking and illegal drug use were associated with both medium- and high-risk classifications, suggesting a broader impact on SU severity. These results imply that family binge drinking may elevate general vulnerability to SU, contributing to moderate risk, while smoking and illegal drug use within the family context may play a greater role in the progression to high-risk patterns. Supporting this interpretation, Rusby et al. (67) found that parental binge drinking predicted earlier initiation of alcohol use in youth—a factor consistently linked to more severe SU outcomes in later adolescence (68). Similarly, longitudinal evidence from Sullivan et al. (69) demonstrated that parental SU increases the likelihood of adolescent SU initiation, further emphasizing the importance of family context in shaping risk trajectories.

Results indicated that sexual activity under SU had the largest association with high-risk group membership compared to all other predictors, underscoring its role as a key etiological factor for problematic SU. This finding aligns with research by Levy et al., (70) who reported a strong association (odds ratio > 5) among U.S. adolescents aged 12 to 18. Specifically, participants with high CRAFFT scores (>2) were significantly more likely than their CRAFFT-negative peers to engage in sexual contact after drinking alcohol, using drugs, or while very high or drunk. Overall, the observed effects of sexual activity under SU may be explained by pre-existing impairments in judgment that exacerbate impulsive and risky behaviors. This explanation is consistent with literature suggesting that alcohol and other substances (e.g., tobacco and marijuana) may impair cognitive functioning, lower inhibitions, increase impulsivity, and reduce individuals’ ability to assess risk accurately (71–74).

Previous exposure to violence (i.e., psychological and sexual) exhibited a similar pattern of association as sexual activity under SU, with an increased likelihood of being in the high-risk CRAFFT categorization compared to low- and medium-risk group membership. This finding is congruent with prior research showing that adverse childhood experiences—such as sexual and psychological abuse—increase the probability of engaging in SU behaviors, including binge drinking, marijuana use, and the use of illegal drugs (75–77). A plausible explanation is that these forms of violence often result in profound trauma, emotional distress, and long-term disruptions to psychological well-being, all of which have been linked to SU as a maladaptive coping mechanism over time (3, 78). These results underscore the importance of incorporating measures of violence exposure into SU prevention efforts targeting adolescents in Costa Rica and similar contexts.

### 4.1 Implications for prevention

Given the substantial proportion of participants in the low-risk group and the limited differences between medium- and high-risk groups—aside from sexual activity under SU and prior exposure to psychological or sexual violence—these findings support the implementation of universal prevention interventions targeting adolescent SU in Costa Rica. While focusing on individuals at high risk remains critical and cost-effective, a major challenge lies in preventing the initial escalation from low- to medium-risk levels. *Universal prevention* approaches may yield substantial public health benefits by shifting social norms and reducing overall substance use risk—a phenomenon known as the “prevention paradox” (79), which suggests that most cases of SU emerge from low-risk populations due to their greater numbers.

In this context, *universal prevention* initiatives in Costa Rica might focus on delaying the initiation of SU, particularly alcohol, tobacco, and marijuana. Early onset has been associated to an increased likelihood of developing SUDs and dependence in adulthood (8, 80), as well as other public health concerns, including cognitive impairment, social impairment (81), and psychological impairment (82, 83). Promoting abstinence during adolescence—or maintaining a low-risk SU status for as long as possible—has been associated with fewer alcohol-related problems during young adulthood (84). In contrast, selective prevention approaches, such as harm minimization strategies that aim to normalize adolescent alcohol use while discouraging excessive consumption, have shown limited or even iatrogenic effects. For example, a longitudinal study comparing two policy contexts—(a) zero tolerance in the United States and (b) harm minimization in Australia—examined whether adolescent drinking patterns predicted alcohol-related problems in young adulthood. Adolescents exposed to harm minimization policies reported higher rates of alcohol use and more permissive family and school norms compared to those in zero tolerance environments. Moreover, early alcohol use was associated with elevated AUDIT scores at age 25, indicating greater alcohol-related harms. The authors concluded that zero tolerance policies were more effective than harm minimization strategies in preventing long-term alcohol-related problems (85).

Costa Rica maintains a zero-tolerance policy for underage drinking and currently implements school-based prevention programs (e.g., *Aprendo a valerme por mí mismo* [Learning to Rely on Myself]). However, given the persistent prominence of alcohol in national culture and public life—as reflected in national survey data indicating that alcohol is the most commonly used substance among Costa Rican students—family-based universal prevention programs appear to be lacking, limiting the country’s ability to implement a comprehensive, multilevel prevention approach. Evidence suggests that improving family communication, reducing family conflict, and fostering strong emotional bonds with parents and peers can serve as protective factors that reduce vulnerability to substance use (86). Costa Rica still lacks evidence-based, culturally adapted, family-level universal prevention programs. Although internationally recognized approaches, such as *Familias Unidas*, *Guiding Good Choices*, *Strengthening Families Program 10–14*, have demonstrated positive outcomes in the family setting (87), there is currently no evidence that these programs have been adapted or evaluated for Costa Rican families.

Strengthening the family-based component of national prevention efforts is crucial, particularly given the central role families play in adolescent development. One promising strategy could involve increasing the national alcohol tax to (a) raise prices and reduce youth access (88) and (b) fund the adaptation, implementation, and evaluation of universal prevention programs at the family and school levels. Such policies could expand access to high-quality programming nationwide while generating local evidence to guide culturally responsive and sustainable public health strategies—reinforcing Costa Rica’s commitment to protecting its youth.

### 4.2 Limitations and future directions

Several important limitations should be considered when interpreting these findings. First, the cross-sectional design restricts causal inference and the ability to establish temporality. Longitudinal studies are needed to clarify these relationships by confirming temporal sequence and causality (89). Second, key variables—including SU within the family, sexual activity under SU, and exposure to various forms of violence—were measured using single-item indicators. Although these captured meaningful associations, they may not fully reflect the complexity, frequency, severity, or context of these experiences. Future national surveys in Costa Rica should incorporate standardized, validated instruments to better assess these constructs, improve interpretability, and facilitate cross-national comparisons.

Third, all data were self-reported, potentially introducing social desirability and recall biases. Participants may have underreported stigmatized behaviors or misremembered past experiences, which could affect the accuracy of the results. Future studies should improve data quality by incorporating objective and multi-source measures. Fourth, because the VI-NSPSSU is the first national survey in Costa Rica to use the CRAFFT, we were unable to retrospectively examine trends in the distribution of adolescent SU risk categories. Future waves of this survey, using the same screening tool, will enable monitoring of trends over time and assessing shifts in the prevalence and patterns of SU risk levels based on CRAFFT. Fifth, as previously discussed, it is important to consider the timing of data collection—surveys were administered in 2021, during the ongoing COVID-19 pandemic, when social restrictions were still in effect in Costa Rica. These circumstances may have influenced participants’ behaviors and social interactions, potentially leading to underestimations of SU and related risk behaviors. Nonetheless, we are confident in the plausibility of our findings, as we were still able to detect associations with key predictors even under conditions of likely underreporting and lower prevalence. We highlight the importance of future nationally representative surveys in Costa Rica continuing to use the CRAFFT screening tool, as doing so will enable longitudinal comparisons and offer valuable insights into whether and how risk patterns have shifted under post-pandemic conditions. This ongoing use could also shed light on the broader impact of pandemic-related restrictions on adolescent SU and associated risk factors.

Finally, we relied on the CRAFFT screening tool’s original threshold for defining high-risk individuals. Although this cut-off has been used consistently across studies—including in Latin American contexts—it may not fully account for cultural or contextual nuances specific to Costa Rica. Future research should be conducted during a period without any social restrictions and consider validating the CRAFFT cut-off point within the Costa Rican population to ensure its appropriateness and accuracy for local use.

## 5 Conclusion

Despite its limitations, this study is the first to use a nationally representative sample in Costa Rica to classify adolescent SU risk levels and identify potential predictors using the CRAFFT screening tool. Older age, SU within the family, sexual activity under the influence of substances, and exposure to psychological and sexual violence emerged as key indicators of SU risk among Costa Rican secondary students. These findings highlight the importance of universal prevention strategies targeting individual, family, and community levels, as such approaches can foster proximal and prosocial developmental environments with the potential to reduce the burden of SU among Costa Rican youth.

## Data Availability

The data analyzed in this study is subject to the following licenses/restrictions: the data analyzed in this study were obtained from the Costa Rican Institute on Alcoholism and Drug Addiction (IAFA) and are not publicly available; access requires formal authorization from IAFA. Requests to access these datasets should be directed to https://iafa.go.cr/sobre-iafa/donde-estamos/#contacto.
